# Supplementary material for: Single Cell Transcriptomic and Chromatin Profiles Suggest Layer Vb Is the Only Layer With Shared Excitatory Cell Types in the Medial and Lateral Entorhinal Cortex
Source: Front Neural Circuits. 2022 Jan 26;15:806154. doi: 10.3389/fncir.2021.806154 (PMC8826650; doi:10.3389/fncir.2021.806154)
Supplement: Supplementary file 14 [file Data_Sheet_1.ZIP › Inh_vs_Exc_Motifs/homerResults/motif6.similar.html]

motif6

## Information for motif6

C
T
G
A
C
T
A
G
T
C
A
G
A
C
T
G
G
A
T
C
G
T
A
C
G
T
C
A
A
G
C
T
A
C
G
T
C
A
T
G
  
Reverse Opposite:  

G
T
A
C
C
G
T
A
T
C
G
A
C
A
G
T
C
A
T
G
C
T
A
G
T
G
A
C
A
G
T
C
G
A
T
C
G
A
C
T
  

|  |  |
| --- | --- |
| p-value: | 1e-29 |
| log p-value: | -6.723e+01 |
| Information Content per bp: | 1.521 |
| Number of Target Sequences with motif | 5801.0 |
| Percentage of Target Sequences with motif | 47.39% |
| Number of Background Sequences with motif | 16320.2 |
| Percentage of Background Sequences with motif | 42.31% |
| Average Position of motif in Targets | 330.6 +/- 257.9bp |
| Average Position of motif in Background | 366.5 +/- 335.5bp |
| Strand Bias (log2 ratio + to - strand density) | 0.1 |
| Multiplicity (# of sites on avg that occur together) | 1.39 |
| Motif File: | file (matrix) reverse opposite |

### Similar de novo motifs found

|  |  |  |  |  |  |  |  |
| --- | --- | --- | --- | --- | --- | --- | --- |
| Rank | Match Score | Redundant Motif | P-value | log P-value | % of Targets | % of Background | Motif file |
| 1 | 0.912 | C T G A C T A G T A C G G T A C G A T C T G C A A G C T A C G T | 1e-15 | -35.791220 | 31.71% | 28.37% | motif file (matrix) |
